# Local Anæsthesia by Congelation

**Published:** 1856-06

**Authors:** H. L. Burpee

**Affiliations:** Williamsburgh, L. I.


### LOCAL ANÆSTHESIA BY CONGELATION.

BY H. L. BURPEE, WILLIAMSBURGH, L. I.

Since my communication on the subject of anæsthesia by congelation, in February last, I have spent much time in investigating, experimenting, and improving upon my apparatus, and consequently have arrived at more definite conclusions upon some points on which, at that time, I could not speak with full confidence.

In the first place, allow me to say that I do not in the least wish to detract from the merits of any person or persons who have taken this matter in hand heretofore; for instance, Dr. Branch, of Galena, Ill., so far as I know, was the first in this country to claim success in extracting teeth without pain, and I trust will receive his due reward. The accounts I have read in this Journal from the pen of J. Richard Quinton, of London, were certainly very interesting and profitable, and, I freely acknowledge, have proved of much service in guiding us in a path where much darkness existed, for I freely confess to former attempts which proved abortive.

So far as Dr. Branch is concerned, the profession at large are greatly in the dark; but I must think that a direct application of cold for the extraction of teeth cannot come into general use, in consequence of the severe pain it causes when applied to a vital tooth, or to one suffering from a high state of inflammation, although the time required to produce numbness is a half minute shorter by such a process. Would such an application answer for general use, an apparatus could be constructed at a trifling expense; but it is with such means that we are in danger of paralyzing the muscles and parieties of the face.

I have now adopted into general practice, an apparatus with which the requisite numbness to extract a tooth without pain can be produced in a length of time varying from one and a half to two and a half minutes, according to circumstances, and this, too, by commencing with warm water, and *graduating the cold at will*, by which process the patient suffers actually nothing in the application, and seldom any pain is felt in removing the tooth. *Perfection*, however, is not claimed. This apparatus consists of a *combination force pump*, by which two fluids (warm and cold) can be thrown together or separately through a flexible tube into a mouth-piece, covering the tooth and surrounding gum, (the muscles of the face and tongue being protected by a *non-conductor*), and passing off through a flexible tube leading to a vessel placed in a convenient position to receive the waste fluid. The length of time employed in preparing for the operation occupies but a few minutes, and the patient is highly pleased with the result. But I most heartily accord with the views expressed by Quinton, that "you must not only graduate the cold on, but must graduate it off;" and herein consists the safety in the use of cold; for although you may protect the muscles of the face and the tongue by having a mouth-piece made of material which will not communicate the cold where you do not wish it to go, there is still danger of pain or hemorrhage, (and sloughing, if the parts be frozen), unless proper precautions are used to prevent it.

In my own practice I have not experienced the slightest difficulty; but allow me to add, I have used *constant* caution.

The cold fluid which I have used is of a nature from which no harm can result, in case of accident or rupture of the mouth-piece.

I am not sure that all the precautions used are always necessary after extraction, and in one instance of direct application of the cold fluid, no serious pain was experienced; but the teeth to which the cold was applied were dead stumps. Hence we may, in some instances, apply cold at once, but it is better to be safe, as the extra trouble is but little.

It is the deliberate conviction of myself and those to whom these facts are known, that this new and powerful agent may be brought into general use for dental surgery, (also in minor operation of operative surgery), in such a manner as greatly to enhance the interest of the dental profession, inasmuch as the " danger to life and limb " is as nothing when compared to ether and chloroform, and its results are quite as satisfactory, so far as freedom from pain is concerned, and its effect on the general health decidedly beneficial, inasmuch as the patient is relieved of much *dreaded* pain and *anxiety of mind*, which often enfeeble persons of slender constitution and poor general health, and render them unfit for their usual avocations for some time subsequent to the removal of the offending matter.

This, then, is the proper view of the matter, that it saves the patient much fatigue of mind as well as body, and leaves *none* of the ill effects of ether and chloroform; and who that can find the requisite fee would not rather pay a treble amount to be relieved from pain in tooth extraction, and know that no danger from any source is to be apprehended.—*New York Dental Recorder.*

